# Aptasensors for Rapid Detection of Hazards in Food: Latest Developments and Trends

**DOI:** 10.3390/bios15090629

**Published:** 2025-09-21

**Authors:** Anjie Guo, Yuan Zhang, Meifeng Jiang, Li Chen, Xinrong Jiang, Xiaobo Zou, Zongbao Sun

**Affiliations:** 1 Department of Food & Biological Engineering, Jiangsu University, Zhenjiang 212013, China; 2 The Quality Monitoring Center for Food and Strategic Reserves of Zhenjiang City, Zhenjiang 212001, China

**Keywords:** aptasensor, food safety, electrochemistry, fluorescence, colorimetry

## Abstract

The presence of hazardous substances in food poses a serious threat to our health. It is important to develop fast, convenient, and inexpensive assays for on-site sensitive analysis of various hazards in food. With the emergence and popularization of aptamers and biosensors, aptasensors have gradually become one of the most important detection techniques for substances such as nucleic acids and small molecules. This paper reviews the recent research progress in the field of aptasensor based on different technologies (such as electrochemistry, fluorescence, colorimetry, among others) for the rapid detection of hazards (such as foodborne pathogens, mycotoxins, pesticides, among others) in food. In addition, the current challenges of different aptasensors are described for the readers, and the future direction of aptasensors is envisioned by comparing the different technologies in order to develop a more suitable aptasensor. This review will not only promote the advancement of aptasensors but also their practical application in daily life to safeguard human health and food safety.

## 1. Introduction

Food safety incidents in daily life have raised a great deal of concern, and food safety issues threaten our health. Ingestion of food contaminated with foodborne pathogens as well as mycotoxins can cause diarrhea, vomiting, and even life-threatening conditions [1,2,3]. Ingestion of harmful residues in food, such as pesticides, heavy metals, and illegal additives, can have serious consequences, including headaches, skin rashes, vomiting, and carcinogenesis [4,5,6]. Therefore, enhanced monitoring of these contaminants is necessary. The commonly used detection methods available for these hazards include plate technology, GC-MS, AAS, and HPLC, among others [7,8,9,10]. Although these methods have a certain degree of sensitivity and reliability, they rely on instrumental operation, which makes the detection process time-consuming and the handling of the operation complicated. It is necessary to study new rapid, sensitive, inexpensive, and simple methods for monitoring hazards in food.

Aptamers are widely used in fields such as drug delivery and biosensing [11]. Aptamers are short, single-stranded DNA or RNA oligonucleotides obtained through an in vitro evolutionary method called SELEX (systematic evolution of ligands by exponential enrichment) [12,13]. This process involves iterative rounds of selection and amplification to enrich sequences with high affinity and specificity for a target molecule [14], enabling them to specifically bind targets such as proteins, small molecules, cells, and tissues [15,16]. The combination of aptamers with electrochemical, fluorescent, and colorimetric sensors has unique advantages over recognition methods such as enzymes, antibodies, and molecular blotting. First, aptamers inherently discriminate between structural analogs, such as mycotoxins and congeners [17]. This confers good specificity and immunity to interference and enhances the signal response of the sensor. Secondly, the aptamer can be modified with -SH, -NH_2_, or Biotin, etc., such as immobilized on the surface of electrodes, magnetic nanoparticles, etc., through Au-S [18], affinity-biotin [19], etc., to reduce the cost and simplify the structure of the sensor [20]. Meanwhile, the aptamer sensor (aptasensor) is resistant to high temperature and other environments, which is suitable for the detection of targets in complex matrix environments and can enhance the stability of the sensor [21]. Therefore, aptasensor has a wide range of applications in the field of detection of hazards in food [22,23,24,25,26,27]. Here, we summarize the aptamer sequences commonly used in this paper, as shown in Table S1 [28,29,30,31,32,33,34].

Studies have been conducted to review the aptasensor in the fields of medicine and the environment [35,36,37,38]. There are fewer related reviews in the field of food hazards detection, and current studies have discussed aptasensors from the perspectives of different food contaminants [39], multifunctional aptasensors for food analysis [40], different SELEX methods, and different aptamers [41,42]. However, in the field of food safety, there are no articles that provide a more comprehensive overview of aptasensors from the perspective of different technological means for the time being. It is important to compare the current research on aptasensors using different technical means and how these aptasensors accomplish the detection of hazards in food, which will provide some direction and guidance for the development of faster methods and more practical portable instruments in the future, and promote newer aptasensors to play an important role in the field of food safety.

**Scheme 1:**
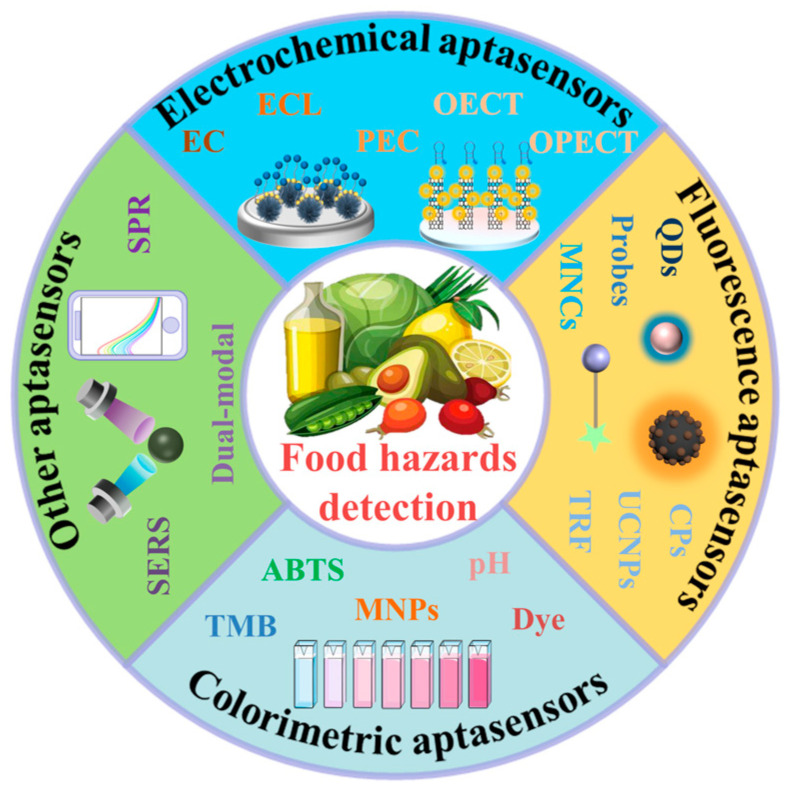
Application of aptasensor based on different novel technologies in the detection of hazards in food.

This article discusses the research progress of aptasensors including electrochemistry (EC), fluorescence (FL), colorimetry (CM) and surface-enhanced Raman spectroscopy (SERS) in the field of rapid detection of foodborne pathogens, mycotoxins, pesticides, heavy metal residues and other hazards in food (Scheme 1), focuses on the mechanism and performance of these aptasensors, summarizes the possible challenges and opportunities of these sensors, and looks forward to future development direction of aptasensor in the detection of hazards in food.

## 2. Electrochemistry-Based Aptasensor Application for the Detection of Hazards in Food

**Figure 1:**
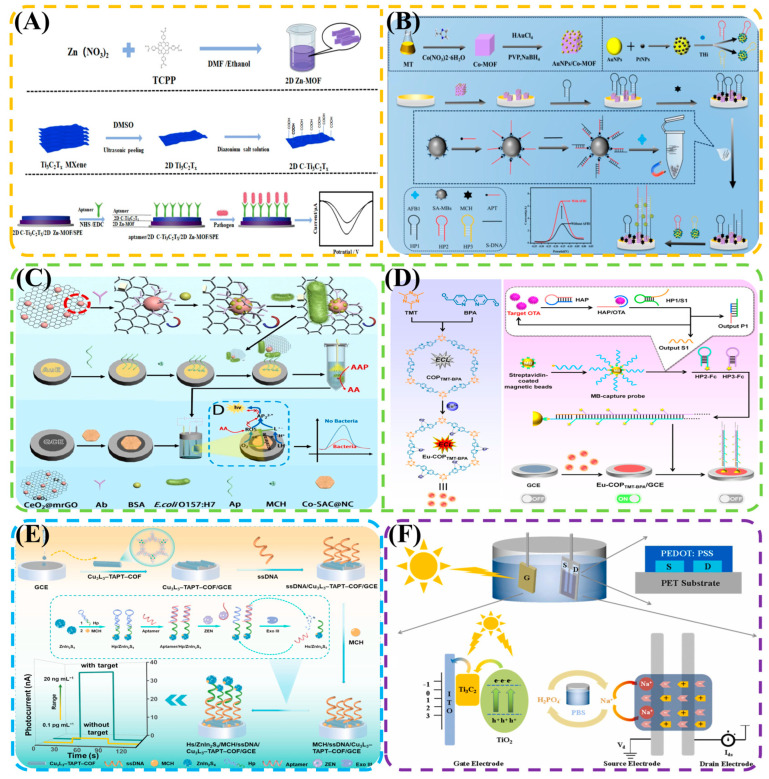
Basic electrochemical aptasensor: (**A**) Based on Apt/2D Ti_3_C_2_T_x_/2D Zn-MOF for detection of multiple foodborne pathogens; (**B**) Based on AuNPs/Co-MOF and THi/Au@PtNPs for detection of mycotoxin AFB1. ECL aptasensor: (**C**) Based on Co-SAC@NC for detection of *E.coli O157/H7*; (**D**) Based on Eu-COP_TMT-BPA_ for detection of OTA. PEC aptasensor: (**E**) Based on TAPT-COF/Cu-N_2_ for the detection of ZEN. OECT/OPECT aptasensor: (**F**) Based on Ti_3_C_2_/TiO_2_ for the detection of Ciprofloxacin.

In recent years, electrochemistry (EC) sensing has risen rapidly in the field of food hazards detection due to its fast response, low cost, and easy miniaturization [43]. With the advancement of nanomaterials, microfluidics, EC sensors are gradually moving toward high sensitivity, multi-target detection, and portability [44]. Traditional biorecognition elements (e.g., antibodies, enzymes) have defects such as poor stability, high cost, and complex modification, which limit the practical application of sensors, while the addition of aptamers gives these sensors a good development space [45]. The following section mainly introduces the basic EC, electrochemiluminescent (ECL), photoelectrochemistry (PEC), and organic photoelectrochemical transistors (OPECT), and discusses the whole EC aptasensor application and development trend by analyzing its principle, construction, and practical application (Figure 1).

### 2.1. Basic EC-Based Aptasensor

EC aptasensors are based on the aptamer capturing the redox reaction that occurs between the target and the electrode surface, which is quantitatively analyzed by measuring the resulting changes in electrical signals (e.g., current, electrical impedance, potential, etc.) [46].

Common foodborne pathogens include *Salmonella*, *Staphylococcus aureus* (*S. aureus*), and *Escherichia coli* (*E. coli*), among others [47]. A rapid one-step electrochemical sensor for the detection of these foodborne pathogens was developed by Yang et al. [48]. The recognition element, signal amplifier, and signal tag were integrated on the electrode surface by constructing an aptamer/2D carboxylated Ti_3_C_2_Tx/2D Zn–Metal–Organic Framework (MOF) composite, respectively (Figure 1A). Aptamer capture of pathogens increased the impedance of the electrode surface, leading to a decrease in the 2D Zn-MOF current. The detection limits for *E. coli*, *S. aureus*, and *Salmonella typhimurium* were 6, 5, and 5 CFU/mL. Lin et al. developed a ratiometric electrochemical aptasensor based on GQDs/Cu-MOF nanocomposites for the detection of *S. aureus* [49]. GQDs were synthesized under ultrasound using graphene as a precursor and then combined with Cu-MOF to prepare GQDs/Cu-MOF nanocomposites for providing an output reference signal and probe DNA-ferrocene for generating a response signal. Competition in the presence of bacteria resulted in probe detachment, and I*_DNA-ferrocene_* diminished I*_Cu-MOF_* increased. The sensitivity of the ratiometric EC aptasensor reached 0.97 CFU/mL.

Mycotoxins are a group of naturally occurring toxic compounds produced by certain types of fungi in agricultural products under specific conditions, including aflatoxin B1 (AFB1), ochratoxin A (OTA), and monotelomeric mycotoxins (DON) [50]. The aptasensors constructed by Yu et al. used enzyme-free Hybridization Chain Reaction (HCR) as a cyclic amplification strategy, highly conductive AuNPs/Co-MOF as the electrode-modified material, and highly catalytically active THi/Au@PtNPs as the signaling tag. Competition in the presence of AFB1 dislodged the activation probe, which was added to the electrode to trigger the HCR, resulting in the generation of a large number of signaling probes, and thus the quantification of AFB1, with a sensitivity of 4.0 × 10^−2^ pg/mL (Figure 1B) [51].

Antibiotics include Oxytetracycline (OTC), kanamycin, etc., which are widely used in veterinary therapy and infection prevention, and may leave residues in animal-derived foods consumed by humans (e.g., milk). Kourti et al. described a preliminary label-free electrochemical aptasensor with antifouling properties to detect OTC in milk samples [52]. The sensor was constructed by modifying a gold screen-printed electrode with α-lipoic acid-NHS and amine-terminated aptamers to quantify OTC in the presence of Fe(CN)_6_^4−^/Fe(CN)_6_^3−^ redox pairs. The detectable concentration range was wide, with a limit of detection (LOD) of 14 ng/mL.

Pesticides include organophosphates, neonicotinoids, etc., and commonly used ones are, for example, acetamiprid (AD) and malathion (ML). Wu et al. constructed aptasensors for the simultaneous detection of AD and ML [53]. Firstly, MB/MOF235 and FcCysAu nanoparticles were designed to be piggybacked on CeMOF (III, IV), which were then connected to the aptamer complementary chains, respectively, to construct signaling markers. Functionalized reduced graphene oxide and NF/HP-UiO66-NH_2_ were synthesized as substrates to load aptamers for AD and ML. When AD or ML was present, the corresponding target–aptamer complexes were formed. Two newly prepared signaling markers were used to assemble the remaining aptamers. The higher the concentration of AD and ML in the sample, the less residual aptamer is present, resulting in fewer signal markers being combined on the electrode surface and thus a lower signal current. The detection limits of this method reached 4.8 pM and 0.51 pM, respectively.

### 2.2. ECL-Based Aptasensor

ECL technology triggers a redox reaction between a luminophore (e.g., luminol, quantum dots, metal nanoclusters) and a co-reactant (e.g., H_2_O_2_, tripropylamine, TPrA) by applying a specific potential to the electrode surface, which generates an excited state intermediate that releases photons when it returns to the ground state [54]. The intensity of the optical signal is inversely or positively proportional to the concentration of the target, thus enabling quantitative detection.

Hu et al. developed an ECL biosensor with a cascade reaction between two nanoenzymes for the detection of *E. coli O157/H7* (Figure 1C) [55]. A cobalt single-atom catalyst (Co-SAC@NC) with oxidase (OD)-like activity acted as a co-reaction promoter for O_2_, catalyzing the conversion of oxygen to reactive oxygen species (ROS) and facilitating the luminal-O_2_ system. CeO_2_@mrGO nano-enzymes were used for labeling. CeO_2_@mrGO captured *E. coli O157/H7*, which was then detected by the aptamer and the antibody’s affinity binding on the electrode surface, forming a sandwich structure on the gold electrode. The ROS were eventually consumed and quenched the ECL emission of the Luminol-O_2_ system. The LOD of the sensor was 2.78 CFU/mL. Tao et al. reported a color-switching ECL based on a dual bipolar electrode (D-BPE) [56]. The D-BPE consists of a cathode filled with a buffer solution and two anodes filled with [Ru(bpy)_3_]^2+^-TPrA and luminal-H_2_O_2_ solutions, respectively. After the introduction of ferrocene (Fc)-labeled aptamers on both anodes, it was difficult to observe an ECL-emitting signal for [Ru(bpy)_3_]^2+^ (anode 1), whereas luminal emitted a strong and visible ECL signal (anode 2). In the presence of foodborne pathogens, the aptamer assembled with them, resulting in the departure of Fc from the surface of the D-BPE anode. Anode 1 increased in intensity while anode 2 emitted a weakened signal. By self-calibrating the ratio of the two signals, the final detection limit was 1 CFU/mL.

Song et al. developed a self-enhanced ECL sensor based on tandem signal amplification for OTA determination (Figure 1D) [57]. Eu-COP_TMT-BPA_ can be used to sensitize Eu^3+^ luminescence. When OTA is present, it binds to the aptamer probe (HAP). The exposed HAP sequence then binds to probe 1 (HP1), releasing the OTA and trigger (S1). The released S1 can be recognized by the magnetic bead (MB) capture probe coupler to trigger the HCR between Fc-labeled HP2 and Fc-labeled HP3, leading to the formation of long double-stranded DNA nanowires on the MB surface and the accumulation of abundant Fc, which allows quenching of ECL intensity. The detection limit LOD of this ECL sensor was 0.47 fg/mL. Xiang et al. designed an ultra-low-potential ECL aptasensor for zearalenone (ZEN) determination based on a resonance energy transfer (RET) system with SnS_2_ QDs/gC_3_N_4_ as a novel luminescent agent and CuO/NH_2_-UiO-66 as a double-bursting agent [58]. SnS_2_QDs were loaded onto gC_3_N_4_ nanosheets and enhanced ECL luminescence by a strong synergistic effect at ultra-low potential. The removal of CuO/NH_2_-UiO-66 from the electrode surface through the binding interaction between the aptamer and ZEN resulted in the inhibition of the RET system and the increase in the ECL signal. The lower limit of detection was 0.085 fg/mL.

Han et al. designed an off–on signal switchable ECL aptasensor for the detection of profenofos. CuO-ABEI-AgNPs were used as ECL enhancers [59]. In addition, a molecular probe, AuNPs-Apt-T, was prepared. In the absence of profenofos, the probe was immobilized at the electrode, resulting in a signal burst (switching to “off”). Instead, it was switched to “on”. Finally, the detection limit of the aptasensor reached 30 ng/mL.

### 2.3. PEC-Based Aptasensor

PEC detection technology is based on the photoelectric effect and requires a light source. Photosensitive materials (e.g., semiconductor quantum dots, metal oxides) generate photogenerated carriers (electrons and holes) when irradiated by excitation light at specific wavelengths. The target interacted with the aptamer to quantify the target concentration by inhibiting or promoting the efficiency of carrier separation and altering the intensity of the photocurrent [60].

Cui et al. constructed a near-infrared (NIR)-driven PEC for the detection and inactivation of *S. aureus* [61]. The SA31 aptamer was immobilized on a PDA/MnO photoelectrode. In the presence of bacteria, it bound specifically to the aptamer, resulting in an attenuated photocurrent signal due to spatial site-blocking effects. A lower limit of detection of 2.0 CFU/mL was achieved. Ge et al. proposed a PEC biosensor coupled with recombinase polymerase amplification (RPA) technology (RPA-PEC) for the detection of a wide range of foodborne pathogens [62]. 3D screen-printed paper-based electrodes were designed with two working surfaces on which *E coli O157/H7* and *Staphylococcus aureus* genomic DNA were triggered by RPA on the corresponding electrode surfaces. The detection limits were 3.0 copies/μL and 7.0 copies/μL, respectively, using the formed DNA-PEC signaling notification genes.

Li et al. developed a novel amplified PEC aptasensor for efficient detection of ZEN (Figure 1E) [63]. Cu_3_L_3_-4,4′,4′′-(1,3,5-triazine-2,4,6-triyl) trianiline (TAPT)-Covalent Organic Framework (COF) contains abundant Cu-N_2_ monatomic sites and was used as both a PEC electrode and a biological platform for anchoring single-stranded DNA. In addition, the p-type ZnIn_2_S_4_ semiconductor anchors the hairpin probe strand hybridized to the ZEN target aptamer. The detection limit was as low as 24 fg/mL.

Ye et al.’s study proposed a PEC extended gate field effect transistor (PEGFET) sensor for the detection of kanamycin [64]. The sensor used ITO glass as the extended gate electrode (photoelectrode) and titanium dioxide as the photosensitive material. The binding of kanamycin to its corresponding aptamer caused the gold nanocluster to catalyze the oxidation of 3,3′-diaminobenzidine (DAB). This interaction led to the precipitation of different amounts of DAB on the surface of the photoelectrode, which resulted in a gate voltage shift and source-drain current response. The final LOD was nM level.

### 2.4. OECT/OPECT-Based Aptasensor

In recent years, OECT/OPECT has been a research highlight in the field of biosensing [65]. OPECT is composed of the photosensitive working electrode in PEC as the gate electrode in OECT, and the principle is that organic semiconductors (e.g., polyaniline, polypyrrole) generate photogenerated carriers under light illumination, and the target combines with the aptamer to change the channel carrier concentration, which in turn modulates the source leakage current (*Ids*) or the threshold voltage (*Vth*) to achieve target quantification [66].

Zhang et al. developed a novel target-induced biped DNA walker-mediated and In_2_S_3_/Ti_3_C_2_(MXene) Schottky junction-gated OPECT sampler [67]. The OPECT-specific detection of the target molecule dibutyl phthalate (DBP) was obtained by alkaline phosphatase-mediated ascorbic acid (AA) enrichment of the In_2_S_3_/MXene photosensitive gate. AA enrichment effectively depleted holes and enhanced the photoelectric response by inhibiting electron-hole pair complexation, resulting in effective modulation of the organic semiconductor. This ultimately provided a low detection limit of 0.18 fM. You et al. constructed an OPECT sensor using a Ti_3_C_2_/TiO_2_ composite as the gate photoactive material (Figure 1F) [68]. Compared with Ti_3_C_2_, TiO_2_, and physically mixed Ti_3_C_2_/TiO_2_, the Ti_3_C_2_/TiO_2_ composite had a larger capacitance, and the OPECT intensity was three times higher than that of Ti_3_C_2_ and two times higher than that of physically mixed Ti_3_C_2_/TiO_2_ and TiO_2_. The detection limit was 1 ng/L with Ciprofloxacin (CIP) as the target.

Ding et al. constructed a sensitive OECT-PEC biosensor for the detection of the organophosphorus pesticide ML [69]. The sensor used poly(3,4-ethylenedioxythiophene) (PEDOT)-modulated iron–metal–organic framework (Fe-MOF) nanocomposites as the photoactive gate material, and poly(3,4-ethylenedioxythiophene)/poly (styrenesulfonic acid) (PEDOT/PSS) as the channel material. The final LOD was 0.03 ng/L.

### 2.5. Brief Summary of the Whole EC-Based Aptasensor for the Detection of Hazards in Food

Table 1 provides a comparative summary of the performance of various EC aptasensors, expanding upon the examples discussed in the previous sections and including additional notable studies for a broader context [70,71,72,73,74,75,76,77,78,79,80,81,82,83,84,85,86,87,88,89,90,91,92,93]. The current status and future development of total EC technology based on the contents of [43,44,45,46,47,48,49,50,51,52,53,54,55,56,57,58,59,60,61,62,63,64,65,66,67,68,69] and Table 1 extensions were briefly discussed. EC is more mature and still the main force of on-site rapid detection; ECL combines the dual advantages of chemiluminescence and electrochemistry detection, with low background noise, high sensitivity, wide dynamic range, etc., and it is irreplaceable in trace detection; PEC has the advantages of extremely low background noise, high sensitivity, self-cleaning ability, etc., and provides new ideas for complex matrix analysis through the optical–electrical synergistic mechanism; OPECT has the advantages of flexible compatibility, low toxicity, and dual-signal output, etc., and leads the development of portable devices with its flexible and environmentally friendly features. Comparatively, EC aptasensor needs to rely on new materials to improve the anti-interference ability and multi-target detection performance; PEC sensors cannot amplify weak signals, and the detection of small currents requires highly sensitive reading devices, which increases the cost of the application, and PEC requires a light source; OPECT has high material requirements and a relatively complex structure.

In summary, the development of nanomaterials has the most important impact on the overall EC sensor sensitivity [94,95]. Functional nanomaterials have properties such as enhanced specific surface area, increased electrode attachment, improved conductivity, and photochemical functionality. Innovations in materials drive the development of an electrochemical aptasensor. The development of aptamers equally affects the performance of the electrochemical aptasensor. Aptamer optimization techniques can solve the problem of insufficient specificity or affinity, and emerging aptamer screening techniques can also lead to more practical aptamer sequences and extend the detection range of aptasensors [96].

Due to the limitations of EC instrumentation, current research has focused on the construction of single-channel sensors. To improve the detection efficiency and enhance the fault-tolerance of the sensing technology, the development of multichannel aptamer array sensors is a future research direction [97]. Meanwhile, the miniaturization of electrochemical aptasensors and their transformation to practical applications are two important challenges that need to be solved, and the combination of microfluidic technology and smartphones is a better solution at present. In conclusion, the electrochemical aptasensor is gradually moving towards high sensitivity, multi-target detection, and portability.

## 3. Fluorescence-Based Aptasensor Application for the Detection of Hazards in Food

**Figure 2:**
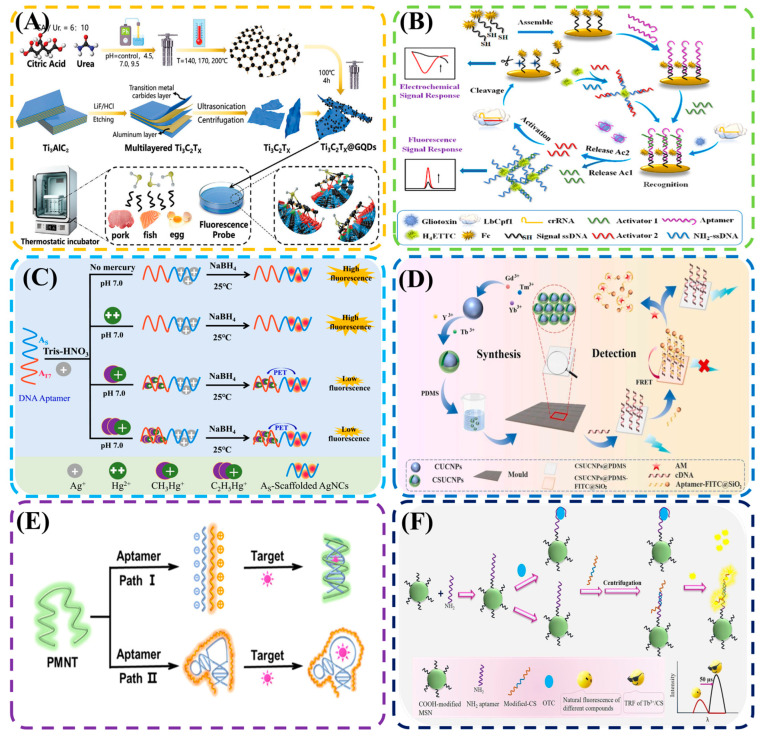
QD fluorescence aptasensor: (**A**) H_2_S detection based on Ti_3_C_2_T_x_ MXene/GQD. Fluorescent Probe aptasensor: (**B**) Based on AIE and CRISPR for gliotoxin. MNC fluorescent aptasensor: (**C**) Based on DNA/ AgNCs to detect organic mercury. (**D**) Fluorescent aptasensor based on UCNPs to detect acrylamide; (**E**) Fluorescent aptasensor based on CPs to detect K^+^, adenosine, cortisol, and caffeine; (**F**) TRF-based aptasensor to detect OTC.

In recent years, fluorescence technology has rapidly emerged in the field of food hazards detection due to its fast response, simple operation, and stable signal [98]. Fluorescence technology usually relies on fluorescent moieties, dyes, and fluorescent nanoparticles. The design of the fluorescent aptasensor is mainly based on fluorescence signal amplification (FSA), fluorescence resonance energy transfer (FRET), and fluorescence polarization (FP) [99], which can achieve speedy and sensitive rapid and accurate detection of hazards in food by detecting changes in fluorescence signals. Aptamers as recognition elements of fluorescent aptasensor can be built faster using four DNA-based FSA reactions, Rolling Circle Amplification (RCA), HCR, High-Density Co-Hybridization Reaction (HD-CHR), and Strand Displacement Reaction (SDR) [100]. In order to improve the detection sensitivity, quantum dot fluorescence (QDs), organic fluorophore probes, metal nanocluster (MNCs), and other technologies are constantly breaking through performance bottlenecks. The following section focuses on these fluorescent aptasensors and discusses the development and trend of the overall fluorescent aptasensor by analyzing its working mechanism, composition, and practical applications (Figure 2).

### 3.1. QD Fluorescence-Based Aptasensor

Semiconductor nanocrystals (e.g., CQDs, GQDs), with size-tunable fluorescence emission peaks and narrow half-peak widths, can radiate different colors according to their sizes, which is conducive to the simultaneous imaging of multiple fluorophores. Their fluorescence properties enable specific recognition by surface-modified aptamers, which are combined with mechanisms such as FRET or fluorescence burst to detect the target. The excellent photochemical stability and specificity of the QD-aptasensor have been used in the field of detection of hazards in food [101].

Li et al. reported a dual-emission CQD-based aptasensor [102], which realized the simultaneous detection of AFB1 and OTA by signal amplification of the CRISPR-Cas12a/Cas13a system, with detection limits as low as 3.1 pg/mL and 3.5 pg/mL, respectively, and the work promoted the integration of CRISPR signal amplification technology with fluorescence aptasensor fusion application. Zhang et al. constructed a ratiometric fluorescent aptasensor for streptavidin (STR) detection [103]. The red fluorescence was derived from AgNCs-SMP@ZIF-8, and the green fluorescence was provided by aptamer-modified CQDs. When STR was present, Cu^2+^ penetrated ZIF-8 to quench the AgNCs’ red fluorescence, while the CQDs’ green fluorescence remained stable, and the detection limit was achieved by ratiometric signal changes as low as 0.98 nM. The study provided a reference for the mechanism of fluorescence dual-signal correction.

Jia et al. developed a 2D/0D heterojunction fluorescent probe based on Ti_3_C_2_T_x_ MXene-loaded GQDs for the rapid detection of H_2_S in food products (Figure 2A) [104]. The aptamer interacted with H_2_S, which inhibited the intramolecular charge transfer effect, recovered the photo-induced electron transfer, and triggered fluorescence bursting, accompanied by a change in color. The detection limit of this work was as low as 41.82 ppb, providing a new method for ultrafast visualization of aptasensor detection. A fluorescence-opening aptasensor for S-GQD was developed for the detection of the organophosphorus pesticide oxomorph (OM) [105]. The aptamer bound to S-GQD and induced fluorescence burst by aggregation; when OM was present, the aptamer bound to OM and restored its fluorescence signal. The method provided a reference for switch-mode sensors.

Yang et al. developed a fluorescence sensing platform based on multilayered Nb_2_C-MXene nano-quenchers with carbon dot-labeled aptamers (B-CDs@Apt) for sensitive detection of antibiotics [106]. The target triggered the release of the aptamer to recover the fluorescence signal. The detection limit of this sensor for chloramphenicol (CAP) was 0.360 ng/mL. This work was the first to apply MXene materials to a paper-based fluorescent aptamer sensing platform, which significantly enhanced the sensitivity. A multicolor fluorescent probe was combined with paper-based microfluidics (mCD-μPAD) for the first time [107], and a “fluorescence off” probe (CD-apt-MoS_2_) was constructed. FRET was used to achieve signal modulation, enabling the rapid quantitative analysis of a variety of antibiotics within 15 min. This study provided a new solution for multi-target antibiotic screening in the field.

### 3.2. Organic Fluorophore Probe-Based Aptasensor

Organic fluorophores include fluorescent probes FAM, Cy series, AMC, TAMRA, and FITC, among others. Detection is mainly achieved by fluorescence burst-recovery or conformational change-induced signaling switches [108].

Zhang et al. developed a fluorescence polarization (FP)-based aptasensor that introduced FAM, whose FP signal was significantly enhanced when free rotation was restricted [109]. When *Salmonella* was present, the target bacteria triggered a cyclic isothermal strand displacement amplification reaction, which captured the FAM into a supramolecular DNA monolayer and dramatically enhanced the FP signal by restricting fluorophore rotation. The method had a low detection limit of 7.2 CFU/mL and provided a FP sensing scheme without burst fluorescence. A dual-signal aptasensor based on the synergistic interaction of AIE and CRISPR-Cas12a was developed for the ultrasensitive detection of gliotoxin (Figure 2B) [110]. When the target was present, the complex dissociated to release the activator, which triggered the aggregation of ETTC-dsDNA to generate AIE fluorescence signals, and the activation of the CRISPR system cleaved ssDNA-Fc to generate an electrical signal. The detection limit was as low as 2.4 fM, successfully overcoming the problem of weak signals of traditional sensors. Ge et al. achieved the simultaneous detection of AFM1 and AFB1 by the FRET mechanism [111]. When the target bound to the aptamer, it triggered the dissociation of the DNA double-crossover structure and released the fluorescent probe, restoring the fluorescent signals of Cy3 (568 nm) and Cy5 (660 nm). The detection limits of this sensor were as low as 6.24 pg/mL and 9.0 pg/mL, respectively, and the dual fluorescence provided a reference for simultaneous detection of dual targets. Amalraj et al. developed an aptasensor based on a dual fluorescence-labeled probe (FAM/TAMRA) with CuO@PDA-MoS_2_ nanospheres for simultaneous detection of Hg^2+^ and CAP [112]. Using the FRET mechanism, the nanospheres quenched the fluorescence signal. When Hg^2+^ was present, probe cleavage was triggered to release FAM, and CAP bound to the remaining ssDNA-TAMRA, releasing TAMRA and generating dual fluorescent signals. The detection limits of this sensor were as low as 86 pM and 45 pM, respectively. The advantages of this sensor were that it simplified the traditional assay process and was cost-effective.

### 3.3. MNCs Fluorescence-Based Aptasensor

MNC materials such as AuNCs, AgNCs, CuNCs, etc., which carry fluorescent properties themselves, are ultrasmall nanoparticles (<2 nm) consisting of several to hundreds of atoms in the transition state from the atomic state to plasma metal nanoparticles [113].

A fluorescent aptasensor based on the synergistic interaction of DNA nanoflowers (DNFs) with AuNCs was investigated for the detection of AFB1 [114]. Combined with Mn-MOF-catalyzed localized hairpin assembly (LCHA), which triggered a conformational change of the hairpin DNA (H1) in the presence of AFB1, generating a large number of amplification products, H2-H3, which, together with the DNF@AuNCs hybridization with DNF@AuNCs, burst the fluorescent signal. The detection limit of the sensor was as low as 7 pg/mL. The design of the sensor cleverly integrated the programmability of the DNF structure, the fluorescence property of AuNCs, and the catalytic amplification function of Mn-MOF. A modulated DNA/AuNCs fluorescence switch aptasensor was developed for in situ detection of OTA in grains [115]. When OTA was absent, Apt-OTA hybridized with the DNA template and burst AgNCs fluorescence; when OTA was present, it bound to the aptamer and prevented hybridization of complementary sequences, and fluorescence was restored. The detection limit of the sensor was 1.3 nM, and it was easy and inexpensive to operate. A DNA/AgNCs-based fluorescent aptasensor without labeling was developed for the detection of organic mercury in seafood (Figure 2C) [116]. In the absence of organic mercury, Ag^+^ reduces on the aptamer sequence to form strongly fluorescent AgNCs. When organic mercury was present, its binding aptamer triggered phototransfer of electrons (PET), which burst the fluorescence of AgNCs. The method had a detection limit as low as 5.0 nM, which required no labeling and was easy to operate.

### 3.4. Upconversion Fluorescent, Conjugated Polymer Fluorescent, and Time-Resolved Fluorescent-Based Aptasensor

Upconversion fluorescent nanoparticles (UCNPs) achieve near-infrared light excitation and visible light emission through rare earth elements (e.g., Yb^3+^/Er^3+^) to avoid autofluorescence interference of biological samples, and have high photostability without a photobleaching problem [117]. For example, Rong et al. designed a method to encapsulate UCNPs in polydimethylsiloxane (PDMS) [118], coupled fluorescein isothiocyanate (FITC) with an aptamer and immobilized it on the surface of the PDMS, and quenched the luminescence of the UCNPs by FRET (Figure 2D). Preferential binding to the aptamer when the target acrylamide was present led to the separation of FITC from UCNPs and restoration of the luminescent signal. The detection limit was 1.00 nM, and its solid-state design combined stability and portability.

Conjugated polymer (CPs) has a signal amplification effect (“molecular wire” effect) through an intramolecular π-π conjugated structure. Characterized by a large Stokes shift, they can avoid excitation light interference. For example, the study by Zhang et al. proposed a label-free aptamer detection method based on cationic CPs (CCPs) (Figure 2E) [119]. By exploiting the conformational changes triggered by aptamer binding to the target, the fluorescence response of the mode CCP material could be modulated to reflect the presence of the target. The study validated its specific detection of K^+^, adenosine, cortisol, and caffeine. The method had broad applications both as a tool for the assessment of aptamer binding capacity and for expansion into label-free biosensors.

Time-resolved fluorescent (TRF) materials include lanthanide complexes such as Eu^3+^ and Tb^3+^ chelates. They are characterized by long fluorescence lifetime (microseconds) and elimination of short-lived background fluorescence by the time-gating technique. It is suitable for complex matrix (e.g., milk, meat) detection. For example, a previous study developed an aptasensor based on TRF technology for hygromycin (OTC) detection (Figure 2F) [120]. The long fluorescence lifetime property of Tb^3+^ was utilized in combination with functionalized mesoporous silica nanoparticles (MSN) to construct the system. The aptamer acted as a recognition element, the modified complementary chain acted as an antenna ligand by enhancing the TRF signal of Tb^3+^, and the MSN served as both substrate support and separation and enrichment. The detection limit of the study was as low as 2.1 nM, and the proposed TRF signal amplification strategy provided a new paradigm for the detection of multiple targets in complex samples.

### 3.5. Brief Summary of the Fluorescence-Based Aptasensor for the Detection of Hazards in Food

Table 2 provides a comparative summary of the performance of various FL-based aptasensors, expanding upon the examples discussed in the previous sections and including additional notable studies for a broader context [121,122,123,124,125,126,127,128,129,130,131,132,133,134,135,136,137,138,139,140,141,142,143,144]. The current status and future development of total FL technology based on the contents of [98,99,100,101,102,103,104,105,106,107,108,109,110,111,112,113,114,115,116,117,118,119,120] and Table 2 extensions were briefly discussed. QD aptasensor suffers from the problems of low quantum yield of GQD and CD fluorescence and potential toxicity of metal QDs [145]. Organic fluorophore probes have the characteristics of easy modification and a flexible burst-recovery mechanism. Disadvantages are susceptibility to photobleaching and low interference resistance; MNC has the properties of size-dependent fluorescence, low toxicity, and easy synthesis. Aptamers can be used as both templates and stabilizers to regulate nanocluster synthesis; UCNPs have the advantages of no background noise and deep tissue penetration, but the sensor innovation is more complicated.

In summary, the fluorescent aptasensor has some limitations. Comparing the data in Table 1, there is an issue with a slightly lower detection limit than that of the electrochemical aptasensor. There are also problems of material toxicity and background influence. Relatively, the advantages of the fluorescence aptasensor are also obvious, such as easy visualization, easy construction, and signal stability, among others. Therefore, the development trend of fluorescence aptasensors is also obvious. Multimode fusion to enhance sensitivity, such as combining fluorescent probes with SERS to acquire fluorescence and Raman signals simultaneously [146]; intelligent design to optimize sensor structure, such as AI-assisted screening of aptamer sequences or optimization of fluorescent material–aptamer coupling efficiency [147]; green development to expand the application scenarios, such as the development of heavy-metal-free quantum dots (e.g., InP QDs) and biodegradable conjugated polymers etc. [148]; portable integration toward practical applications, such as fluorescent aptasensors coupled with smartphone spectrometers for on-site rapid detection [149].

## 4. Colorimetry-Based Aptasensor Application for the Detection of Hazards in Food

**Figure 3:**
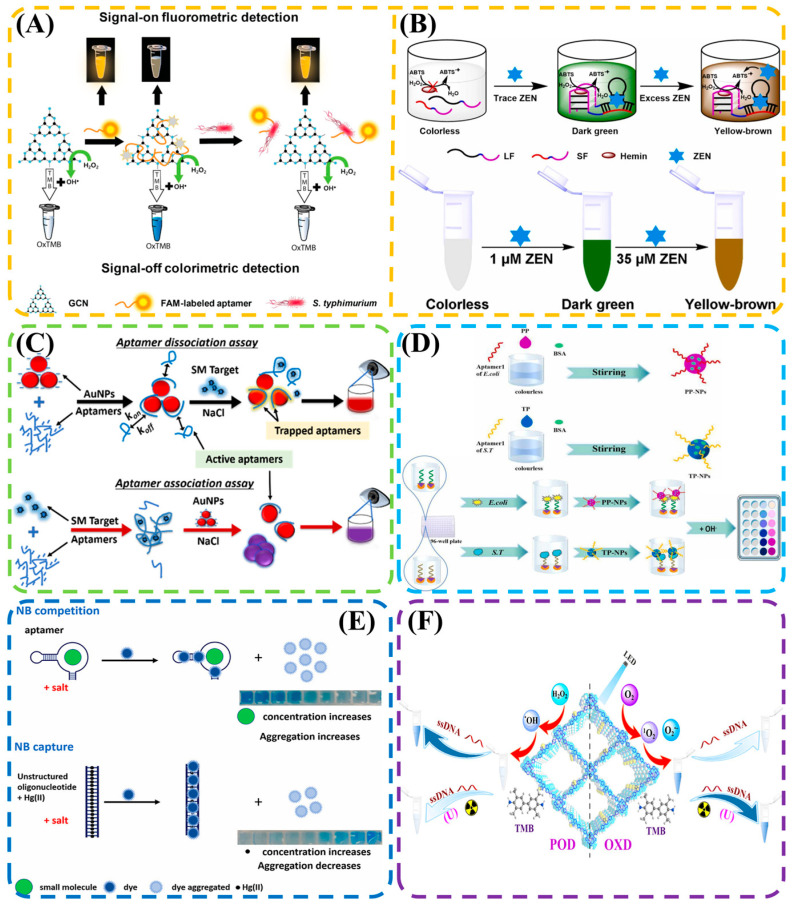
TMB/ABTS chromogenic aptasensor: (**A**) Based on GCN for the detection of *Salmonella typhimurium*; (**B**) Based on splitting aptamers for the detection of ZEN. Metal nanoparticle chromogenic aptasensor: (**C**) Based on AuNPs to detect methamphetamine. (**D**) pH-based color rendering aptasensor to detect *E.coli* and *Salmonella typhimurium*. (**E**) Dye-based color rendering aptasensor to detect Hg^2+^. (**F**) Stimulus-responsive material-based aptasensor to detect UO_2_^2+^.

Aptamer-based colorimetric methods mainly involve the use of catalytic color development, such as nanoparticles and enzymes, to produce structural colors as output signals for the detection of analytes [150]. Compared with other detection methods, colorimetric methods have the advantages of visualization, simplicity, and rapidity, and are suitable for on-site analysis and real-time detection (Figure 3) [151].

### 4.1. TMB/ABTS Color Development-Based Aptasensor

The oxidation of TMB by horseradish peroxidase (HRP), nanoenzymes (e.g., Fe_3_O_4_NPs, G4/hemin), etc., catalyzes the generation of a blue product (maximal absorption peak at 652 nm) and thus collects the color signal for analysis [152].

Dang et al. studied the development of a graphitic carbon nitride (GCN)-based aptasensor for the detection of *Salmonella typhimurium* (Figure 3A) [153]. In the presence of the target bacteria, the peroxidase-like activity of GCN was inhibited due to aptamer dissociation, and TMB color development was diminished. Conversely, GCN catalyzes the blue coloration of TMB. The detection limit was as low as 8 CFU/mL, and no complicated labeling or amplification steps were required, which made it inexpensive. Au@Fe_3_O_4_ NPs were used to compose the nano-enzyme and modify the aptamer [154]. In the absence of *E. coli*, the nano-enzymes utilized peroxidase-like activity to oxidize TMB to produce a blue color, and vice versa, the blue color diminished. The lower limit of detection was 3 CFU/mL. Fe-N-C monoatomic enzymes (SAzymes) were combined with CRISPR/Cas12a technology to construct a colorimetric aptasensor for the detection of AFB1 in grains for the first time [155]. When AFB1 was absent, the aptamer bound to crRNA to activate Cas12a enzyme activity, which cleaved single-stranded DNA and released SAzymes, catalyzing the blue coloration of TMB; conversely, it reduced the coloration signal. The sensor had a low detection limit of AFB1 as low as 0.01 ng/mL, and also provided theoretical guidance for the design and mechanism study of single-atom enzymes.

Similar to the principle of TMB-based color development, ABTS is catalyzed by HRP or nanoenzymes, etc., to oxidize to produce a green product (maximum absorption peak at 405 nm), which collects the color signal for analysis.

A vision/smartphone dual-mode aptasensor based on exonuclease III (Exo III)-assisted signal amplification with G-quadruplex DNAzyme was investigated for rapid detection of *Staphylococcus aureus* [156]. The aptamer specifically recognized the bacterium and triggered the Exo III cyclic reaction, releasing a large amount of heme/G-quadruplex DNAzyme, which catalyzed the oxidation of ABTS to produce a green product, ABTS•−, with the intensity of color development proportional to the concentration of the bacterium. The sensor had a detection limit as low as 32 CFU/mL, and in combination with a smartphone, enabled immediate detection (POCT) without specialized equipment. Lai et al. developed a two-color optical sensor based on a split aptamer for the detection of ZEN (Figure 3B) [157]. In the absence of ZEN, the aptamer separated from the DNAzyme fragment, and the solution was colorless. When ZEN was present, it bound to the aptamer to activate the catalytic activity of DNAzyme and oxidized ABTS to dark green ABTS•+, and when the concentration of ZEN was too high, it further reacted with ABTS•+ to generate a yellow-brown product. The sensor had a detection limit of 6 nM and achieved a two-color gradient response. A dual-channel ratiometric colorimetric aptasensor based on CeO NCs was developed for the detection of microcystin-LR [158]. CeO NCs possessed a quadruple enzyme mimetic activity, and the MC-LR aptamer was utilized to modulate the peroxidase activity of CeO NCs, which produced an inverse response to the substrates TMB and ABTS. The sensor had a low detection limit of 0.66 pg/mL, providing a reference for dual-signal self-calibrating assays.

### 4.2. MNPs Color Development (AuNPs, AgNPs) Aptasensor

Gold and silver nanoparticles (Au/AgNPs), etc., can exhibit color (red and yellow, respectively) through the localized surface plasmon resonance (LSPR) effect. The aptamer binds to the target and modulates the aggregation or dispersion state of the nanoparticles, which causes a change in the solution color (e.g., from red to blue when AuNPs are aggregated) [159].

Chang et al. reported a colorimetric sensor based on AuNPs with split aptamers for the detection of estradiol [160]. The method was based on the target-induced recycling assembly of split aptamer fragments. Upon addition of the target, the aptamer bound to estradiol and regenerated in the presence of helper DNA, forming a tee-like junction (3 WJ) structure, which significantly enhanced the salt-induced aggregation effect of AuNPs and triggered a change in solution color (red to blue). The sensor, with a detection limit as low as 0.7 nM, was simple and sensitive. A colorimetric aptasensor based on peptide-capped cationic AuNPs was developed for the detection of bisphenol A (BPA) [161]. The BPA aptamer adsorbed onto the surface of AuNPs via electrostatic interaction. The aptamer preferentially bound to and detached from BPA when BPA was present. The reduction of free aptamer led to the aggregation of AuNPs under high salt conditions, and the solution color changed from red to blue. The detection limit was 87.04 pM, and no complex labeling or instrumentation was required, which was inexpensive. Sen et al. revealed that the adsorption of aptamer on the surface of AuNPs would gradually increase with time to form a more stable conformation, thus reducing their dynamic responsiveness to the target (Figure 3C) [162]. It was also proposed that by adjusting the addition order of the target, aptamer, and AuNP in the detection system, the sensitivity of the sensor for methamphetamine detection could be significantly improved, and the rapid detection of the target in oral fluid could be realized. This study provided a new direction for the rational design of LSPR aptasensors.

### 4.3. Other Colorimetric Aptasensors: pH, Dye, and Stimulus-Responsive Material-Based Color Development

The principle of a pH-based aptasensor is that the aptamer binds to the target and triggers a chemical reaction (e.g., DNAzyme cleavage of the substrate releases H^+^), which alters the solution pH and reveals the color by phenol red, bromocresol green, and other pH indicators. A Visual detection was achieved by constructing aptamer-modified pH-responsive nanoparticles using pH indicator color changes triggered by specific binding of *E. coli* and *Salmonella typhimurium* to the aptamer (Figure 3D) [163]. The combination of a colorimetric sensor and the sandwich method amplified the signals to reach the lower limit of detection of 1 CFU/mL for both pathogens. The method integrated a robotic arm and NEMO software control system to realize one-button operation, which provided a reference for fully automated food contaminant monitoring.

The principle of the dye-based aptasensor is that the aptamer acts as a “gating molecule” to control the release of the dye (e.g., SYBR Green I embedded in a DNA double-strand) or encapsulation (e.g., adsorption of the dye by graphene oxide) [164], and the binding of the target to the aptamer disrupts the gating structure, releases the dye, and develops the color. A salt aggregation-based colorimetric aptasensor was proposed (Figure 3E) [165]. Using Nile blue (NB) as a dye, the addition of salt enhanced the aggregation ability of NB and changed its absorption spectrum, while the aptamer inhibited NB aggregation by binding to double-stranded DNA or nucleobases. When the target was present, it competed for the conjugation of the aptamer with NB, leading to NB aggregation and triggering a change in the color of the solution from blue to colorless. This method successfully realized the Hg^2+^ detection.

Stimulus-responsive materials develop color, e.g., temperature-/photosensitive materials (e.g., poly(N-isopropylacrylamide), azobenzene derivatives) undergo phase or conformational changes upon aptamer–target binding, causing a change in solution turbidity or color. Zhang et al. developed a colorimetric sensor based on a photosensitive covalent organic framework (Tph-BT) nano-enzyme for the detection of uranyl ions (UO_2_^2+^) (Figure 3F) [166]. At its core, it utilized an inverse regulatory mechanism of the aptamer on the activity of Tph-BT nanoenzymes, in which the aptamer bound to the target to form a secondary structure and detached from the surface of Tph-BT in the presence of UO_2_^2+^, restoring the oxidase activity (catalyzing the TMB to show blue color) and inhibiting the peroxidase activity (catalyzing ABTS to show green color attenuation). This study opened up new avenues for the development of smart nano-enzyme sensors.

### 4.4. Brief Summary of the Colorimetric-Based Aptasensors for the Detection of Hazards in Food

Table 3 provides a comparative summary of the performance of various CM aptasensors, expanding upon the examples discussed in the previous sections and including additional notable studies for a broader context [167,168,169,170,171,172,173,174,175,176,177,178,179,180,181,182,183,184]. Based on the contents of [150,151,152,153,154,155,156,157,158,159,160,161,162,163,164,165,166] and Table 3 extensions, we briefly discussed the current status and future development of total CM technology. The sensitivity of colorimetric methods is relatively lower than that of EC and fluorescence methods. Meanwhile, the accuracy and stability of colorimetric aptitude sensors can be affected by other substances in the system. Therefore, improving the sensitivity and solving the interference problem will promote its further development. Considering the easier acquisition of colorimetric signals, the development of array sensors for the detection of multiple hazards in food is one of the future research directions. Smartphone-derived colorimetric tools have the potential to revolutionize food safety control. Colorimetric aptasensors based on pH dyes and other colorimetric aptasensors can also be used as indicators, with reference to food freshness monitoring, and here they can be used for food contaminant monitoring.

In summary, some elements of development are proposed here based on the characteristics of colorimetric aptasensors. Multimode fusion, e.g., TMB colorimetry coupled with SERS for simultaneous acquisition of colorimetric and spectroscopic signals [185]; portable device integration, taking advantage of colorimetric visualization in combination with smartphone image analysis for on-site quantification [186]; and innovative material substitution, e.g., development of new catalytic systems to reduce detection costs [187].

## 5. Other Technologies Based on Aptasensor Applications for the Detection of Hazards in Food

### 5.1. SERS-Based Aptasensor

In recent years, Raman spectroscopy and surface-enhanced Raman spectroscopy (SERS) have been widely explored in food safety analysis due to their ultra-high sensitivity, excellent spectral resolution, and unique molecular fingerprinting techniques [188,189]. The SERS aptasensor principle is based on the change in photon energy due to the interaction between the aptamer and the target under laser irradiation, which leads to the quantification of the target [190].

Xiao et al. investigated a SERS-based aptasensor for rapid detection of pathogens [191]. RAuMNPs were used as capture probes for efficient enrichment of target pathogens in complex samples. Aptamers/DTNB/gold nanoparticles were used as SERS labels. The sensor had a low detection limit of 8 CFU/mL for *E. coli O157/H7*, realizing the synergistic effect of magnetic separation and SERS signal amplification.

Jiao et al. presented a SERS-based sensor for the quantitative detection of AFB1 [192]. The core of its design was aptamer-functionalized aminated MSN, and the detection was achieved by target-triggered release of the SERS signaling molecule 4-mercaptophenylboronic acid (4-MPBA). The detection limit of this method was as low as 0.03 ng/mL, which provided a new idea for the rapid analysis of food toxins.

**Figure 4:**
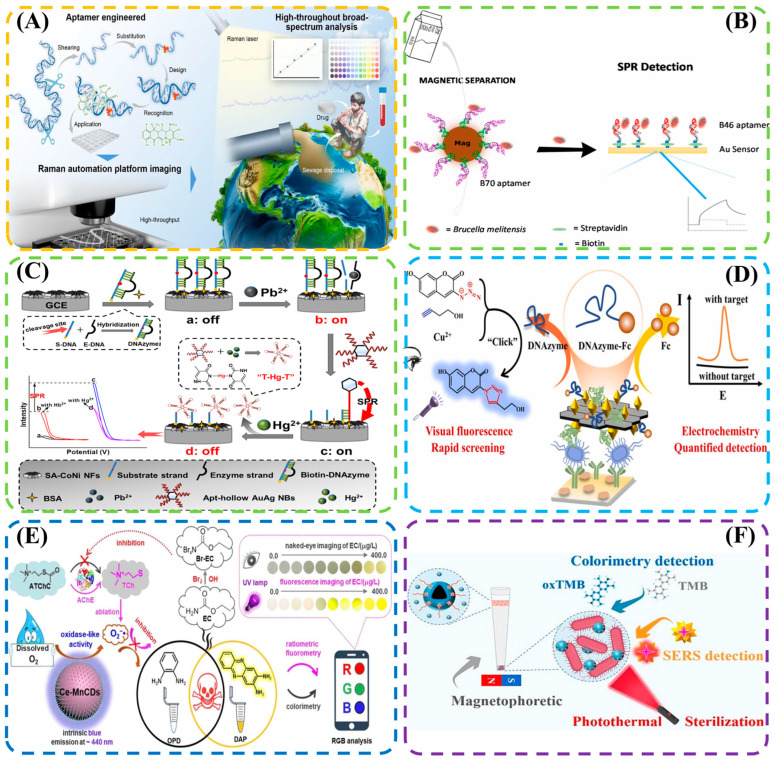
SERS aptasensor: (**A**) Detection of TC based on automated SERS. SPR aptasensor: (**B**) Based on magnetic separation SPR to detect *B. melitensis*; (**C**) Based on AuAg NB to detect Pb^2+^ and Hg^2+^. Aptasensor fused with multiple technologies: (**D**) Based on electrochemistry fluorescence dual-mode detection of VP; (**E**) Based on colorimetry fluorescence dual-mode detection of EC; (**F**) Based on SERS colorimetry dual-mode detection of VP.

SERS may signal instability in complex system detection. Liu et al. constructed a detection system for broad-spectrum analysis by optimizing tetracycline (TC) aptamers and combining an automated platform with SERS imaging (Figure 4A) [193]. Binding to the aptamer triggered a signal response when TC was present, enabling trace detection. The detection limit of this strategy reached 0.07 pM, which provided an innovative SERS sensing and imaging solution for the detection of complex systems through the optimization of aptamers and the combination of multiple technologies.

### 5.2. SPR-Based Aptasensor

SPR-based aptasensors are optical sensing devices that utilize the sensitivity of surface plasma (a special type of electromagnetic field) to change the refractive index, and the aptamer is usually immobilized on a gold layer as an identification probe. The interaction with the target alters the SPR angle to enable quantification [194].

Dillen et al. investigated a fiber-optic surface SPR (FO-SPR)-based biosensor that detected targets through the synergistic interaction of a double-stranded aptamer (DA) and AuNP [195]. Binding to the aptamer in the presence of the target triggered the spatial rearrangement of covalently immobilized AuNP, which significantly amplified the FO-SPR signal through SPR coupling. With a detection limit of 230 nM for single-stranded DNA and high specificity, it broke through the performance of traditional FO-SPR and provided a new strategy for real-time and repetitive detection of drugs, toxins, and other targets.

Dursun et al. developed an aptasensor for the detection of *Brucella melitensis* (*B. melitensis*) based on SPR coupled with magnetic separation technology (Figure 4B) [196]. The aptamer was screened by the bacteria-SELEX technique and immobilized on the surface of the SPR chip. When magnetically separated and purified bacteria were bound to the chip, the SPR signal changed in proportion to the bacterial concentration. The sensor had a low detection limit of 27 ± 11 bacteria/mL and innovatively integrated magnetic separation pre-enrichment with real-time SPR detection.

Au and Ag were used as excellent SPR sources and could also be combined with ECL to improve its performance. Li et al. constructed an ECL aptasensor based on gold and silver nanoboxes (AuAg NB) for the detection of Pb^2+^ and Hg^2+^ (Figure 4C) [197]. Pb^2+^ was detected by DNAzyme-catalyzed cleavage of nucleic acids to generate ECL signals, and Hg^2+^ was inhibited by binding to the aptamer to quench the ECL signals by the SPR process. The sensors achieved detection limits of 0.07 fM and 4.07 pM, respectively, and provided a reference for innovative ECL design.

### 5.3. Aptasensor Based on Integration of Multiple Technologies

The fusion of multiple techniques can effectively improve their performance and even realize the greater effect of synergy. Techniques such as electrochemistry, fluorescence, and colorimetry have been widely used in the field of food hazards detection [198,199,200].

A photo-enhanced electrochemistry (PEEC) and colorimetry dual-mode aptasensor had been developed using rGO-AuNP Schottky contacts for AFB1 monitoring [201]. The PEEC mode allowed for ultrasensitive quantification based on the photo-enhanced electroactivity mechanism, while the colorimetry mode provided rapid threshold level qualitative determination via a portable colorimeter. Highly sensitive and visual detection was achieved at the same time.

Wang et al. presented a biosensing strategy based on the synergistic output of visual fluorescence and electrochemistry dual signals for VP detection (Figure 4D) [202]. DNAzyme-catalyzed click chemistry generated visible fluorescence signals, and ferrocene oxidation reduced EC signals released by MXene/gold nanobipyramid/antimicrobial peptide composite nanoprobes. The detection limit was as low as 6 CFU/mL, providing an all-in-one solution for on-site food testing that combined immediate visual screening with accurate quantitative analysis.

Wu et al. designed a smart instant EC detection device based on Ce –Mn CD materials driven by UV and fluorescence analyses and steady-state kinetics experiments to explore the optical properties and oxidative enzyme-like activities of the materials, thus revealing the mechanism of dual-mode quantitative analysis and sensitively detecting the EC (Figure 4E) [203].

Li et al. developed a dual-mode sensor based on the multifunctional composite magnetic material Fe_3_O_4_@MOF to achieve highly sensitive detection and photothermal sterilization of VP (Figure 4F) [204]. The aptamer efficiently enriched VP and subsequently catalyzed TMB color development using the peroxidase-like activity of the material to achieve the dual functions of detection and sterilization under near-infrared light irradiation via the SERS effect. The detection limits of this strategy were 9 CFU/mL and 7 CFU/mL, respectively, providing an innovative tool for rapid diagnosis and immediate treatment of foodborne pathogens.

Other techniques, such as acoustic sensing, can also be integrated with existing aptasensors. Spagnolo et al. designed an aptasensor to detect *Pseudomonas aeruginosa* in milk samples [205]. The sensor performed mass-sensitive acoustic sensing of bacteria through a thickness-shear mode (TSM) system, and incorporates antifouling technology, incorporating the antifouling linker molecule 3-(2-mercaptoethoxy) propionic acid in the sensing layer (HS-MEG-COOH). The sensor reduced the LOD in milk to 46 CFU/mL. The low quality and rapid sensitive detection demonstrate the aptasensor’s ability to quantitatively identify bacteria in real samples of complex matrices.

### 5.4. Brief Summary of Other Aptasensors for the Detection of Hazards in Food

Table 4 provides a comparative summary of the performance of aptasensors based on SERS, SPR, and integration of multiple technologies, expanding upon the examples discussed in the previous sections and including additional notable studies for a broader context [206,207,208,209,210,211,212,213,214,215,216,217,218,219,220,221,222,223]. And based on the contents of [190,191,192,193,194,195,196,197,198,199,200,201,202,203,204,205] and Table 4 extensions, we briefly discussed the current status and future development of SERS, SPR, and the integration of multiple technologies. SERS aptasensor has ultra-high sensitivity and resistance to photobleaching. However, their performance is extremely dependent on the substrate material. Therefore, there are several future trends. Combining novel substrate materials, for example, developing self-assembled, reusable nanostructures [224,225]; portability and instant detection, for example, combining microfluidics, handheld Raman instrumentation or smartphone for rapid on-site screening [226,227,228]; multimode coupling, such as combining with EC, fluorescence, and AI-assisted techniques to enhance the detection dimensions [229]; combining different signal processing methods, such as ratio signals and “on-off” switching signals, which effectively reduce the signal background and improve detection sensitivity [230,231]. SPR aptasensor does not need to be labeled to allow real-time dynamic monitoring. However, its sensitivity is limited, and the cost of the equipment is high. Miniaturization and integration are the future directions, such as the development of FO-SPR and smartphone-integrated SPR devices [232]. Dual-mode aptasensors provide higher sensitivity and specificity for the detection of hazards in food. However, they are relatively complex to design and fabricate, which can lead to higher costs and data challenges. Future research could consider multimodal integration, miniaturization, and more advanced data analysis techniques to further optimize their application in the field of hazards detection in food [233,234,235,236,237].

## 6. Conclusions and Future Prospects

**Figure 5:**
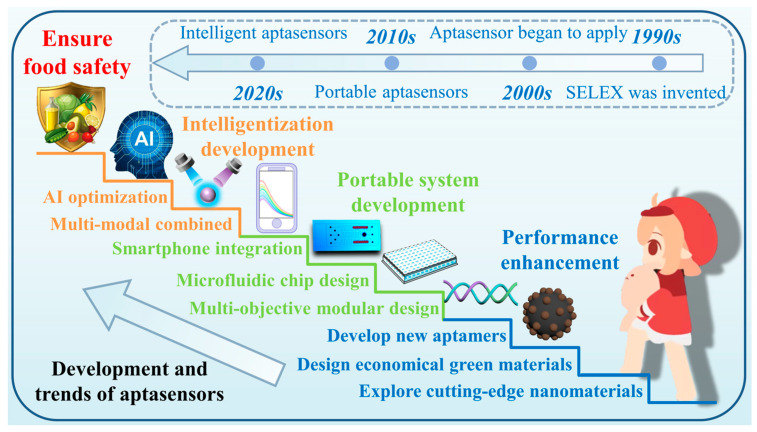
Materials, equipment, and technology of aptasensors: development and trends.

At present, in the field of the detection of hazards in food, the performance requirements of aptasensors are becoming higher and higher, and they are all developing in the direction of being faster and more convenient. This brings a lot of challenges to the innovation in materials, equipment, and technology. For the future development of aptasensors in food testing, several aspects are proposed here (Figure 5). First, the challenges and opportunities of nanomaterials and technologies are addressed. Although nanomaterials (e.g., MOFs, COFs) significantly enhance the sensitivity and functional diversity of aptasensors, they also increase the complexity and cost. In the future, a balance between performance and practicality needs to be sought, e.g., the development of low-cost and reusable nanomaterials. Second, multi-technology integration and intelligence should be considered. For example, multimodal aptasensors can be combined with artificial intelligence, neural networks, and deep learning to optimize aptamer screening, signal data analysis, and sensor design. Moreover, smartphones and microfluidics can be combined in the preparation of highly integrated sensors. Third, aptamers and emerging technologies of synergistic innovation should aim to accelerate the development of novel aptamers, optimize the sequence design to enhance aptasensor specificity and environmental adaptability, and achieve multi-target simultaneous detection through the modular design of multi-aptamer arrays.

In addition, based on the limitations identified in this review (e.g., matrix interference, cost-effectiveness, and field-portability), we propose three forward-looking paradigms: (1) From “Sensing” to “Sencing”: Future nanomaterials should be designed with multifunctionality, incorporating not only recognition and signal transduction but also built-in antifouling properties and self-validation capabilities to ensure reliability in complex food matrices. (2) Closed-Loop Intelligent Diagnostics: The integration of multi-parameter aptasensor arrays with artificial intelligence (AI) and Internet-of-Things (IoT) platforms is crucial. This will enable real-time data analysis, predictive contamination forecasting, and even automated decision-making for food safety intervention, moving beyond mere detection to active risk management. (3) Sustainable and Ubiquitous Monitoring: The development of biodegradable, low-cost, and disposable sensing materials is essential for large-scale deployment. Coupled with the miniaturization enabled by novel manufacturing techniques like roll-to-roll printing, this will pave the way for affordable, ubiquitous food safety monitoring throughout the entire farm-to-fork chain.

At the same time, aptamer quality and repeatability are challenged, and for some aptamers, it is reported that they do not really bind to their intended targets, which will limit the application or even mislead the detection of related substances. Therefore, it is important to select good aptamers for sensor design. Several options are proposed here: (e.g., determination of dissociation constant Kd, selectivity testing for structural analogues, and validation in complex matrices). Advocate for more rigorous reporting standards and validation protocols in academia when presenting novel aptamer sensors. This will lead readers, especially new entrants to the field, to critically evaluate aptamer selection when designing sensors.

As a cutting-edge technology, the development of aptasensors needs to take into account both innovation and practical needs. In the future, through the in-depth integration of materials science, information technology, and bioengineering, aptasensors will break through the existing bottlenecks and realize wider applications in the field of food hazards detection, and truly become the “intelligent sentinel” to guard human health.

## Figures and Tables

**Table 1:** EC-based aptasensor application for the detection of hazards in food.

Aptasensor	Hazards in Food	Nanomaterials	Linear Range	LOD	Reference
EC	*S. aureus*	MWCNTs-Au	1.04 × 10^1^–1.04 × 10^8^ CFU/mL	3 CFU/mL	[70]
	*E. coli*	GOx-AuNPs-COF-H2	10^2^–10^8^ CFU/mL	10 CFU/mL	[71]
	AFB1; OTA	DNA-TDN/HPG	0.05–360 ng/mL; 0.05–420 ng/mL	3.5 pg/mL; 2.4 pg/mL	[72]
	OTA	DNA/Au NCs-PCZIF/hemin	1 pg/mL–500 ng/mL	0.347 pg/mL	[73]
	ML	B-CuO/g-C_3_N_4_	0.18–5.66 pg/mL	1.2 pg/mL	[74]
	AD	DNA-HPG/AuE	0.5–300 nmol/L	0.34 nmol/L	[75]
ECL	*S. aureus*	Arg/ATT-AuNCs	1.0 × 10^1^–1.0 × 10^9^ CFU/mL	1.16 CFU/mL	[76]
	*E. coli*	tetraphenylethene (TPE) derivatives	10–10^7^ CFU/mL	1.99 CFU/mL	[77]
	DON	Ti_3_C_2_ dots/Ti_3_C_2_ nanosheet	0.001–20 ng/mL	0.3 pg/mL	[78]
	OTA	Au-P/Ag NCs	10^−5^–10^−10^ mg/mL	1.36 × 10^−11^ mg/mL	[79]
	AD	Fe-MIL-101@ABEI@AuNPs	1 × 10^−3^ × 10^2^ nmol/L	0.3 pmol/L	[80]
	hydrogen peroxide	ssDNA/g-C_3_N_4_ NS	0.1 fmol/L–10 mmol/L	33 amol/L	[81]
PEC	VP	Bi_2_S_3_/GO@Cu_2_O	1.0–1.0 × 10^6^ CFU/mL	1.0 CFU/mL	[82]
	*E. coli*	graphene oxide-MoS_2_	(1.0–25.0) × 10^7^ CFU/mL	2.0 CFU/mL	[83]
	Patulin (PAT)	CdTe QDs/Au NRs	50 fg/mL–500 ng/mL	30 fg/mL	[84]
	ZEN	ZnO-NGQDs	1.0 × 10^−13^–1.0 × 10^−7^ g/mL	3.3 × 10^−14^ g/mL	[85]
	AD	BiOI/APWE	1 fmol/L–20 nmol/L	0.73 fmol/L	[86]
	ML	CPBI@UCNP/NiMn-LDH/CdS	0.01 ng/L–5 μg/L	4.8 fg/L	[87]
OECT/OPECT	AFB1	Chitosan-graphene	0.01–100 fg/mL	0.01 fg/mL	[88]
	T-2 toxin	ZnO	100 pg/L–1 mg/L	28.8 pg/L	[89]
	Okadaic acid	MXene@SnO/Ce-MOF	0.1 nmol/L–100 µmol/L	42.9 pmol/L	[90]
	Okadaic acid	Cd_0.5_Zn_0.5_S/ZnIn_2_S_4_ QDs	100 pmol/L–0.5 μmol/L	12.5 pmol/L	[91]
	Tobramycin	ZnIn_2_S_4_/TiO_2_	0.1 pmol/L–100 nmol/L	0.18 pmol/L	[92]
	ofloxacin	CdZnS/S-MXene	1.0 × 10^–13^–1.0 × 10^–6^ mol/L	3.3 × 10^–15^ mol/L	[93]

**Table 2:** FL-based aptasensor application for the detection of hazards in food.

Aptasensor	Hazards in Food	Nanomaterials	Linear Range	LOD	Reference
QDs	*E. coli; S. aureus; S. typhimurium; L. monocytogenes; P. aeruginosa*	CsPbBr_3_/PQDs	1.0 × 10^3^–1.0 × 10^7^ CFU/mL	94–136 CFU/mL	[121]
	*Salmonella*	MBs/QDMs	3–3 × 10^6^ CFU/mL	2 CFU/mL	[122]
	ZEN	NGQDs-apt/CdTe QDs@SiO_2_	0.32–320 pg/mL	0.32 pg/mL	[123]
	ZEN	CdTe QDs/WS2 NTs	0.1–100 pg/mL	0.1 pg/mL	[124]
	ML	CQDs/GNPs	1 × 10^−9^–1 × 10^−2^ mol/L	0.13 × 10^−9^ mol/L	[125]
	Okadaic acid (OA); Saxitoxin (STX)	S, P-GQDs/OVA-AuNPs	2.5–128.0 ng/mL; 2.5–29.5 ng/mL	1.8 ng/mL; 0.6 ng/mL	[126]
Organic fluorophore probes	*S. typhi*; *S. aureus*	FAM	10^0^–10^8^ CFU/mL	1 CFU/mL	[127]
	*S. aureus*	FAM/Eu-MOF	7.9–7.9 × 10^8^ CFU/mL	3 CFU/mL	[128]
	ZEN; FB_1_; OTA; AFB_1_	FAM; HEX; ROX; Cy5	0.005–11.11 μg/L; 0.41–100 μg/L; 0.005–3.70 μg/L; 0.015–33.33 μg/L	4 pg/mL; 0.483 ng/mL; 6 pg/mL; 13 pg/mL	[129]
	FB_1_	AIE	1 pg/mL–100 ng/mL	0.89 pg/mL	[130]
	Atrazine	TFT/G4	0.01–50 μg/L	0.25 pg/mL	[131]
	Omethoate	Cy3/GO	0–750 nmol/L	0.16 nmol/L	[132]
MNCs	Foodborne pathogens	DMSN@AuNCs@SiO_2_	10–10^6^ CFU/mL	3 CFU/mL	[133]
	*Cronobacter sakazakii*	AuNCs/G4	1.10 × 10–1.10 × 10 CFU/mL	1.10 × 10 CFU/mL	[134]
	ZEN	AuAg NCs	0.02- 0.625 ng/mL	0.017 ng/mL	[135]
	OTA; AFB1	AuNCs	0.05–200 ng/mL	6.7 pg/mL; 8.6 pg/mL	[136]
	Hg^2+^; Cu^2+^	AuAg NCs/ENM	1–100 μmol/L	12.36 nmol/L; 25.90 nmol/L	[137]
	Triazophos	AuNCs/ZIF-8	0.1–1000 ng/mL	0.07 ng/mL	[138]
UCNPs, CPs, TRF	*E. coli O157/H7*	UCNPs	10^5^–10^8^ CFU/mL	10^5^ CFU/mL	[139]
	*Salmonella*	TRF	10^2^–10^6^ CFU/mL	84 CFU/mL	[140]
	ZEN; OTA	UCNPs	0.5–100 ng/mL; 0.1–50 ng/mL	0.44 ng/mL; 0.098 ng/mL	[141]
	Patulin (PAT)	OA-UCNPs	0.1–1 ng/mL	5.3 pg/mL	[142]
	Hg^2+^	EBSUCNPs/PDANPs	0.5–20 μg/L	0.28 μg/L	[143]
	Carbendazim (CBZ)	UCNPs-MnO_2_	0.1–5000 ng/mL	0.05 ng/mL	[144]

**Table 3:** CM-based aptasensor application for the detection of hazards in food.

Aptasensor	Hazards in Food	Nanomaterials	Linear Range	LOD	Reference
TMB, ABTS	*S. aureus*	HRP-UCNPs-cDNA	56–5.6 × 10^6^ CFU mL	20 CFU/mL	[167]
	*E. coli*	G3/Hemin	1.3 × 10^3^–1.3 × 10^7^ CFU/mL	1.3 × 10^3^ CFU/mL	[168]
	AFB1	HRP@DNA	0.001–350 ng/mL	8 pg/mL	[169]
	AFB1	CdS/UiO-66	5 pg/mL–50 ng/mL	9.5 pg/mL	[170]
	Fipronil	ZIF-8	0.2–4 μmol/L	0.036 μmol/L	[171]
	Tetrodotoxin	Fe_3_O_4_@Cu	0.5–1000 ng/mL	0.243 ng/mL	[172]
MNPs	*E. coli O157/H7*	AuNPs	1.2 × 10^2^ CFU/mL–9.0 × 10^3^ CFU/mL	147.6 CFU/mL	[173]
	AFB1; OTA	Fe_3_O_4_@GO; Fe_3_O_4_@AuNPs	5–250 ng/mL; 0.5–80 ng/mL	-	[174]
	ZEN	PDDA/AuNPs	2.5–100 ng/mL	0.98 ng/mL	[175]
	Sulfadimethoxine	AuNPs	0.2–5 ppm	0.023 ppm	[176]
	T-2 toxin	AuNPs	0.1 ng/mL–5000 ng/mL	57.8 pg/mL	[177]
	melamine (MEL)	AuNPs	0.1–100 μmol/L	42 nmol/L	[178]
pH, dye, stimulus-responsive material	*E. coli*	Metalloporphyrin dye/pH	-	-	[179]
	ZEN	HA-DNA/MOFzyme	0.001–200 ng/mL	0.8 pg/mL	[180]
	Aflatoxin	NO_2_BDP@MOF; HBDP@PSN; COOCH_3_-Diol@PSA	-	-	[181]
	AFB1	Polystyrene	0.05–1 μg/mL	4.56 ng/mL	[182]
	Trimethylamine	PVDF-anthocyanins	20–160 μmol/L	2.52 μmol/L	[183]
	Estradiol	Isoquinoline alkaloids	0.5–5 μmol/L	326 nmol/L	[184]

**Table 4:** Other aptasensor applications for the detection of hazards in food.

Aptasensor	Hazards in Food	Nanomaterials	Linear Range	LOD	Reference
SERS	*S. aureus*	Au@Ag NPs	28–2.8 × 10^6^	0.25 CFU/mL	[206]
	*S. aureus*	Au@NTP@SiO_2_	36.0–3.6 × 10^8^ CFU/mL	2.0 CFU/mL	[207]
	ZEN	MSN-Rh6G-AuNPs	3–200 ng/mL	0.0064 ng/mL	[208]
	PAT	GO@Au	1–70 ng/mL	0.46 ng/mL	[209]
	CBZ	AuNS/Ag/PVDF/CQD	0.002–10 μmol/L	0.86 nmol/L	[210]
	Chlorpyrifos (CPF)	AuNS@4-MBN@Ag	2.5 × 10–5.0 × 10 pg/mL	220.35 pg/mL	[211]
SPR	CAP	Ag/BiOI/TiO_2_	1 nM–250 nmol/L	0.27 nmol/L	[212]
	AFB	MXene/AuNPs	0.01–100 μg/kg	15 μg/kg	[213]
	brevetoxin B	Au	0.05 nM–2000 nmol/L	0.8 nmol/L	[214]
	BSA	AuNPs	1 ng/mL–10 mg/mL	19.46 ng/mL	[215]
	17β-estradiol (E2)	IO-SnO_2_/Au NPs	15 pM–30 nmol/L	0.33 pmol/L	[216]
	Enrofloxacin (ENR)	Au/Bi_24_O_31_Br_10_	0.72–36,000 ng/L	0.30 ng/L	[217]
FL/CM dual-mode	*L.monocytogenes*	Pt NPs	10^1^–10^6^ CFU/mL	10 CFU/mL; 38 CFU/mL	[218]
PEC/EC dual-mode	*Salmonella Enteritidis*	Bi_4_NbO_8_Cl/In_2_S_3_	1.5 × 10^2^–1.5 × 10^7^ CFU/mL	12.9 CFU/mL; 12.3 CFU/mL	[219]
EC/CM dual-mode	AFB1	Au/Ni-Co LDH NC	0.2–100 ng/mL; 50–100 ng/mL	0.071 pg/mL; 18.6 pg/mL	[220]
FL/SERS dual-mode	OTA	AuNSs/AuNPs	1–100 ng/mL; 5–250 pg/mL	0.17 ng/mL; 1.03 pg/mL	[221]
FL/CM dual-mode	Ofloxacin (OFL)	MSN/TMB	0.1–1000 μg/kg; 0.3–1000 μg/kg	0.048 μg/kg; 0.165 μg/kg	[222]
FL/CM dual-mode	CPF	UCNPs-Fe/Zr-MOF	0.05–500 ng/mL	0.028 ng/mL; 0.043 ng/mL	[223]

## Data Availability

No primary research results, software, or code have been included, and no fresh data were generated or analysed as part of this review.

## Supplementary Materials

The following supporting information can be downloaded at: https://www.mdpi.com/article/10.3390/bios15090629/s1, Table S1: Targets, sequences and Kd values of key aptamers used in food hazards detection.

## Abbreviations

The following abbreviations are used in this manuscript:

Apt	Aptamer
SELEX	Systematic evolution of ligands by exponential enrichment
EC	Electrochemistry
ECL	Electrochemiluminescent
PEC	Photoelectrochemistry
OPECT	Organic photoelectrochemistry transistors
FL	Fluorescence
CM	Colorimetry
SERS	Surface-enhanced Raman spectroscopy
*S. aureus*	*Staphylococcus aureus*
*E. coli*	*Escherichia coli*
AFB1	Aflatoxin B1
OTA	Ochratoxin A
DON	Monotelomeric mycotoxins/deoxynivalenol/vomitoxin
AD	Acetamiprid
ML	Malathion
ZEN	Zearalenone
LOD	Limit of detection
NIR	Near-infrared
RPA	Recombinase polymerase amplification
MB	Magnetic bead
AA	Ascorbic acid
CIP	Ciprofloxacin
FRET	Fluorescence resonance energy transfer
QDs	Quantum dots
MNCs	Metal nanoclusters
UCNPs	Upconversion fluorescent nanoparticles
CPs	Conjugated polymers
TRF	Time-resolved fluorescent
TMB	Tetramethylbenzidine
ABTS	2,2′-azino-bis(3-ethylbenzothiazoline-6-sulfonic acid)
SPR	Surface plasmon resonance
