# Supplementary material for: Aptasensors for Rapid Detection of Hazards in Food: Latest Developments and Trends
Source: Biosensors (Basel). 2025 Sep 21;15(9):629. doi: 10.3390/bios15090629 (PMC12467590; doi:10.3390/bios15090629)
Supplement: Supplementary file 1 [file biosensors-15-00629-s001.zip › biosensors-3839116-supplementary.pdf]

Supporting Information

Aptasensors for Rapid Detection of Hazards in Food: Latest Developments and Trends

Anjie Guo [1], Yuan Zhang [1], Meifeng Jiang [1], Li Chen [1], Xinrong Jiang [2], Xiaobo Zou [1] and Zongbao Sun [1,*]

1. *Department of Food & Biological Engineering, Jiangsu University, Zhenjiang, 212013, China*
2. *The Quality Monitoring Center for Food and Strategic Reserves of Zhenjiang City, Zhenjiang, Jiangsu 212001, China*

*Corresponding author, *zongbaos@163.com*

Table S1. Targets, sequences and Kd values of key aptamers used in food hazards detection.

Targets	Aptamer sequence	Kd value	Reference
E. coli	ATTTCGCCCCCGTGTTCCGACTGGTATCTTCACG TCTTCGAGTGT	3.9 nM	Kim et al. (2020)
S. typhimurium	CAGTCCAGGACAGATTCGCGAGGCACAATCCA CCTCTCACCGCACGCCACGCACTGCCTCTGTCC CGCACGTGGATTTCATTCAGCGATT	19.63 nM	Wang et al. (2017)
Salmonella typhimurium	GGGAGAGCGGAAGCGUGCUGGGCCUAGUGUG AGAGCCGUGAGUGAAAGGCCGCGACAAAGAU CGGA-CAUAACCCAGAGGUCGAUGGAUCCCC	20.3 nM	Han et al. (2013)
OTA	GATCGGGTGTGGGTGGCGTAAAGGGAGCATCG GACA	50 nM	Xu et al. (2022)
AFB1	CCATATGCGTGCTACGAGAGGTCAGATAATGCA CTATGG	2.5 μM	Yang et al. (2022)
Acrylamide	CAGTCCAGGACAGATTCGCGAGTGGTCGTGGT GAGGTGCGTGTATGGGTGGTGGATGAGTGTGTG GCCAGGTGGATTTCATTCAGCGATT	17.2 nM	Hu et al. (2018)
Bisphenol A	TTCGAACACGAGCATGCCGGTGGGTGGTCAGG TGGGATAGCGTTCCGCGTATGGCCCAGCGCATC ACGGGTTCGCACCAGGACAGTA	-	Ahn et al. (2012)

Reference

[1] Kim, H. R., Song, M. Y., and Kim, B. C. Rapid isolation of bacteria-specific aptamers with a non-SELEX-based method. *Analytical Biochemistry*, **2020**, *591*, 113542.

[2] Wang, L., Wang, R., Chen, F., Jiang, T., Wang, H., Slavik, M., and Li, Y. QCM-based aptamer selection and detection of Salmonella typhimurium. *Food chemistry*, **2017**, *221*, 776-782.

[3] Han, S. R., and Lee, S. W. In vitro selection of RNA aptamer specific to Salmonella typhimurium. *Journal of Microbiology and Biotechnology*, **2013**, *23*(6), 878-884.

[4] Xu, G., Zhao, J., Yu, H., Wang, C., Huang, Y., Zhao, Q., and Liu, M. Structural insights into the mechanism of high-affinity binding of ochratoxin A by a DNA aptamer. *Journal of the American Chemical Society*, **2022**, *144*(17), 7731-7740.

[5] Yang, C. H., and Tsai, C. H. Aptamer against Aflatoxin B1 obtained by SELEX and applied in detection. *Biosensors*, **2022**, *12*(10), 848.

[6] Hu, Q., Wang, R., Wang, H., Slavik, M. F., and Li, Y. Selection of acrylamide-specific aptamers by a quartz crystal microbalance combined SELEX method and their application in rapid and specific detection of acrylamide. *Sensors and Actuators B: Chemical*, **2018**, *273*, 220-227.

[7] Ahn, J. Y., Lee, S., Jo, M., Kang, J., Kim, E., Jeong, O. C., and Kim, S. Sol-gel derived nanoporous compositions for entrapping small molecules and their outlook toward aptamer screening. *Analytical chemistry*, **2012**, *84*(6), 2647-2653.
